# A single amino acid substitution in the *Bombyx*-specific mucin-like membrane protein causes resistance to *Bombyx mori* densovirus

**DOI:** 10.1038/s41598-018-25388-7

**Published:** 2018-05-09

**Authors:** Katsuhiko Ito, Kurako Kidokoro, Susumu Katsuma, Hideki Sezutsu, Keiro Uchino, Isao Kobayashi, Toshiki Tamura, Kimiko Yamamoto, Kazuei Mita, Toru Shimada, Keiko Kadono-Okuda

**Affiliations:** 1 Division of Insect Sciences, National Institute of Agrobiological Sciences, Tsukuba, Ibaraki, 305-8634 Japan; 2 Department of Agricultural and Environmental Biology, Graduate School of Agricultural and Life Sciences, The University of Tokyo, Bunkyo-ku, Tokyo, 113-8657 Japan; 3 Department of Science of Biological Production, Graduate School of Agriculture, Tokyo University of Agriculture and Technology, Fuchu, Tokyo, 183-8509 Japan; 4 Transgenic Silkworm Research Center, National Institute of Agrobiological Sciences, Tsukuba, Ibaraki, 305-8634 Japan; 5 Present Address: Division of Biotechnology, Institute of Agrobiological Sciences, National Agriculture and Food Research Organization, Tsukuba, Ibaraki, 305-8634 Japan; 6 Present Address: Division of Applied Genetics, Institute of Agrobiological Sciences, National Agriculture and Food Research Organization, Tsukuba, Ibaraki, 305-8634 Japan; 7 Present Address: State Key Laboratory of Silkworm Genome Biology, Southwest University, Chongqing, 400715 China

## Abstract

*Bombyx mori* densovirus type 1 (BmDV) is a pathogen that causes flacherie disease in the silkworm. The absolute nonsusceptibility to BmDV among certain silkworm strains is determined independently by two genes, *nsd-1* and *Nid-1*. However, neither of these genes has been molecularly identified to date. Here, we isolated the *nsd-1* gene by positional cloning and characterized the properties of its product, NSD-1. Sequence and biochemical analyses revealed that this gene encodes a *Bombyx*-specific mucin-like glycoprotein with a single transmembrane domain. The NSD-1 protein was specifically expressed in the larval midgut epithelium, the known infection site of BmDV. Sequence analysis of the *nsd-1* gene from 13 resistant and 12 susceptible strains suggested that a specific arginine residue in the extracellular tail of the NSD-1 protein was common among susceptible strains. Germline transformation of the susceptible-type *nsd-1* (with a single nucleotide substitution) conferred partial susceptibility to resistant larvae, indicating that the + ^*nsd-1*^ gene is required for the susceptibility of *B. mori* larvae to BmDV and the susceptibility is solely a result of the substitution of a single amino acid with arginine. Taken together, our results provide striking evidence that a novel membrane-bound mucin-like protein functions as a cell-surface receptor for a densovirus.

## Introduction

Densoviruses belong to the family *Parvoviridae* and infect insects and crustaceans^1^. Although histochemical and pathobiological studies have shown that host and tissue specificity vary considerably among densoviruses^2–4^, little is known about the host factors that control host and/or tissue tropisms, such as host entry receptors for densoviruses.

*Bombyx mori* densovirus (BmDV) is a pathogen of flacherie disease in the silkworm larvae. BmDV infects only in the columnar cells of the midgut epithelium, and multiplies in the nuclei of the infected cells. The nuclei become hypertrophic by the amplification of the virus, and then the cells are disrupted^5^. Histopathological studies on the midgut epithelium of silkworm larvae infected with BmDV revealed hypertrophic columnar cell nuclei that stained markedly with the Feulgen reaction or methyl green^5^. Finally, the degenerated columnar cells were observed to be liberated into the midgut lumen. BmDV multiplied only in the nuclei of columnar cells of the midgut epithelium, and no pathological changes were in the goblet cells of the midgut or other tissues^5^.

BmDV was previously classified into type-1 (BmDNV-1) and type-2 (BmDNV-2 and BmDNV-Z) according to differences in virulence against silkworms, serological characteristics, and genomic structures^6–12^. However, BmDNV-2 and -Z were recently excluded from the family *Parvoviridae* because of their bipartite genome structure and the presence of a DNA polymerase motif in their genomes, and have been reassigned to a new family *Bidnaviridae* as BmBDV^13^. Interestingly, certain silkworm strains show high susceptibility to one or both types of BmDV and BmBDV, while others show absolute resistance (Figure S1)^6,7,14^. Four unlinked mutations, *non-susceptibility to BmDNV-1* (*nsd-1*)^15^, *Non-infectious to BmDNV-1* (*Nid-1*)^16^, *non-susceptibility to BmDNV-2* (*nsd-2*)^17^, and *non-susceptibility to Zhenjiang (China) strain of BmDNV* (*nsd-Z*)^18^, each of which causes nonsusceptibility to either BmDV or BmBDV, have been reported. We previously identified the *nsd-2* gene by positional cloning and found that this gene encodes a putative amino acid transporter that may function as a cell-surface receptor for BmBDV^19^. During BmDV infection, *nsd-1* and *Nid-1* block the early and late steps of virus infection in the silkworm, respectively^20^. However, neither of these genes has been molecularly identified to date. Therefore, cloning of both BmDV resistance genes will provide significant information about how densoviruses can infect silkworm midgut cells.

Here we report that the *nsd-1* gene encodes a *Bombyx*-specific mucin-like membrane protein expressed only in the midgut. Through DNA sequencing and germline transformation, we have also demonstrated that a single amino acid in NSD-1 determines the specific interaction between BmDV and silkworm midgut epithelial cells.

## Results

### Positional cloning of the *nsd-1* locus

**Figure 1:**
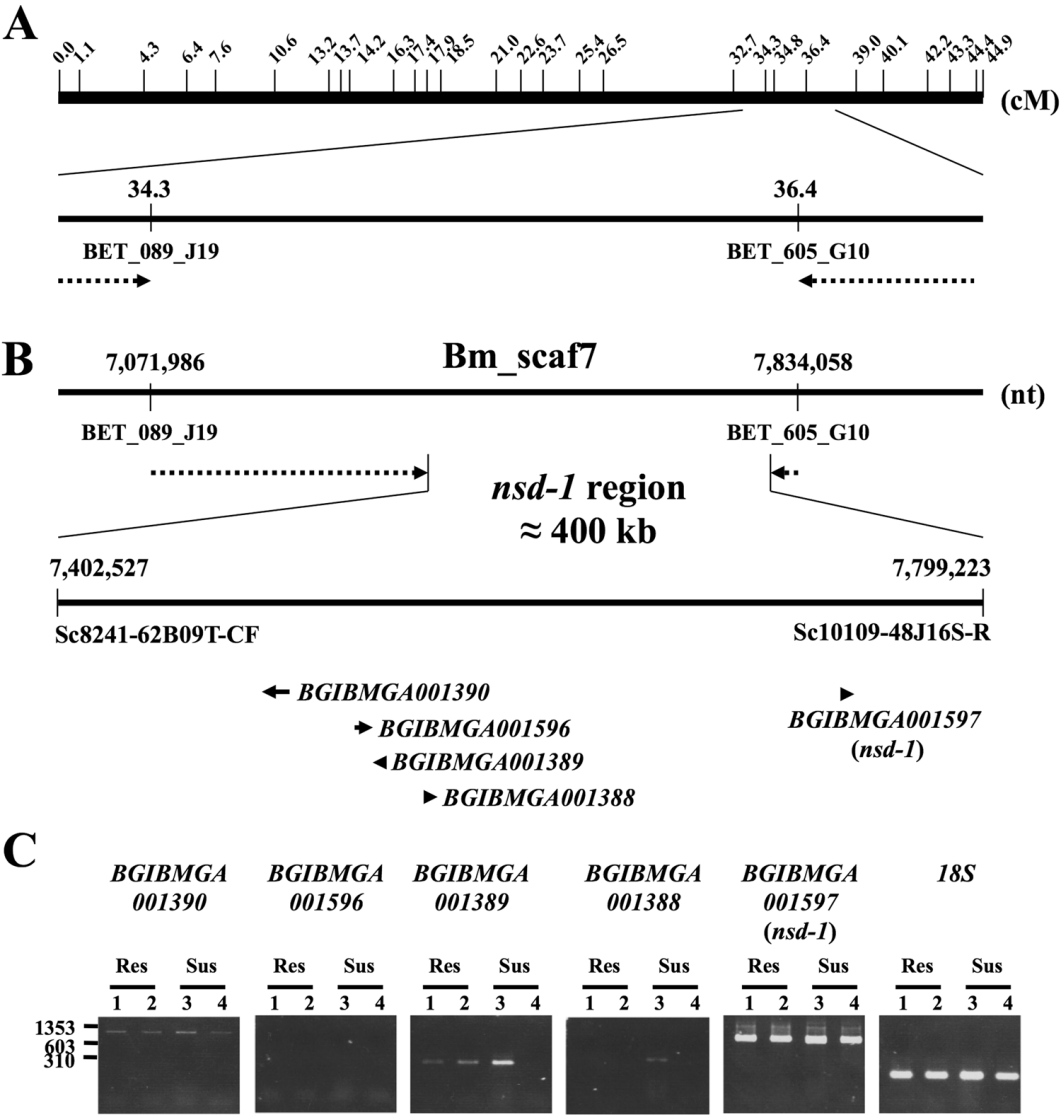
Mapping of *nsd-1* in linkage group 21. (**A**) SNP markers and linkage analysis. The upper panel indicates the positions of the SNP markers in BAC end sequences^21^. Distances between the markers are shown in centiMorgans (cM). In the lower panel, the dotted arrows indicate the results of rough mapping of the region linked to *nsd-1*. (**B**) Fine mapping on the scaffold sequence. The dotted arrows indicate the results of linkage analysis, which was performed to narrow the *nsd-1* region. The *nsd-1* locus was located within an area of approximately 400-kb-long between 7,402,527 and 7,799,223 bp on Bm_scaf7. This region contains five predicted genes: *BGIBMGA001390*, 001596, 001389, 001388, *and 001597*. (**C**) RT-PCR analysis of predicted genes located within the *nsd-1* region in resistant and susceptible strains. The internal control was *18S* ribosomal RNA. Lane 1, C124 (resistant); 2, p50T (resistant); 3, J124 (susceptible); and 4, J150 (susceptible). A mixture of λ/*Hin*dIII and Φ×174/*Hae* III (New England Biolabs) was used as a DNA size marker.

**Figure 2:**
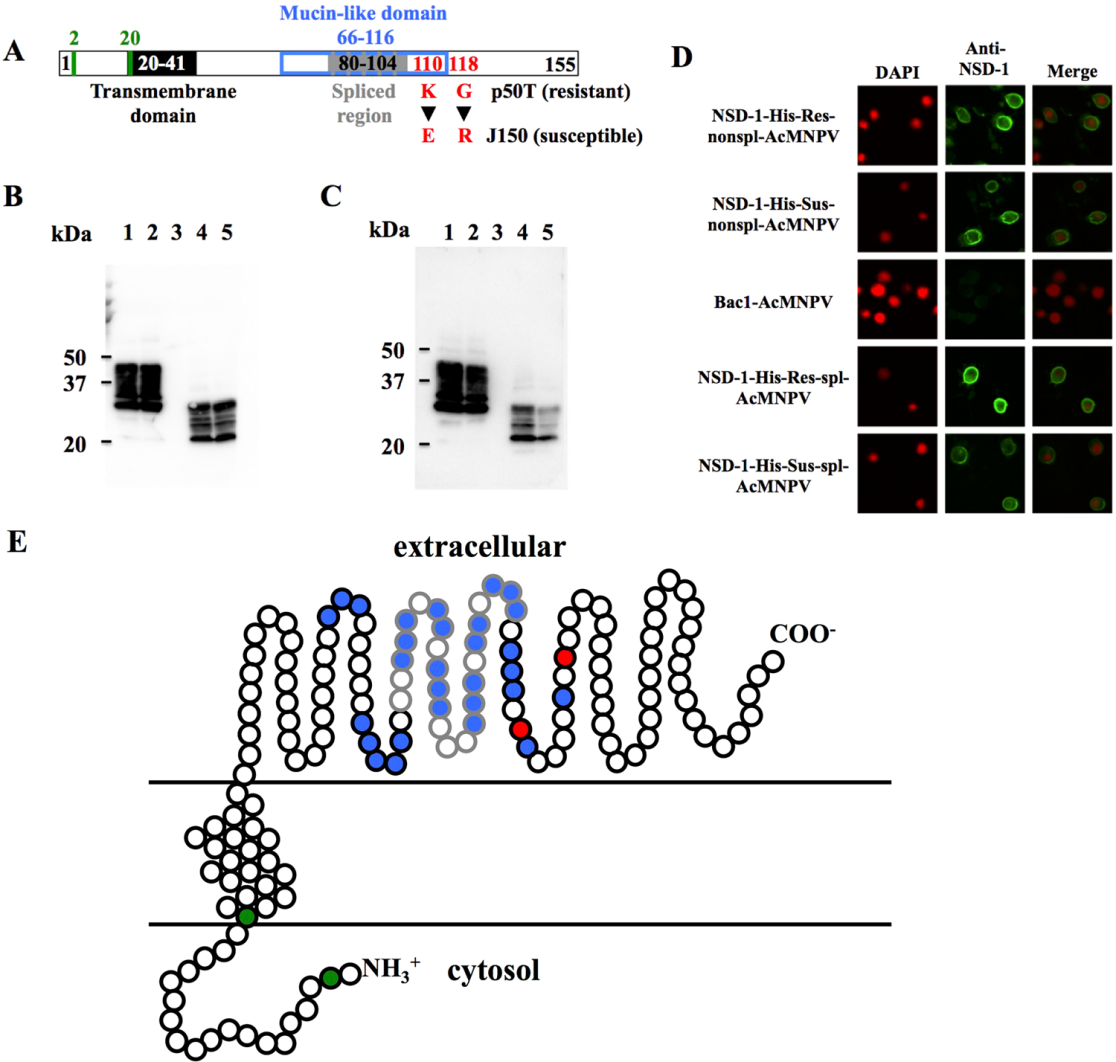
Characterization of the NSD-1 protein. (**A**) Predicted NSD-1 polypeptide. The figure indicates the nonspliced form of NSD-1. Black and gray boxes indicate the transmembrane domain and the region corresponding to the intron in the spliced form of *nsd-1*, respectively. Green and blue boxes indicate the putative *N*-linked and *O*-linked glycosylation (mucin-like domain) sites, respectively. K (lysine) at 110 and G (glycine) at 118 indicate the amino acid residues derived from the resistant strain p50T, while E (glutamic acid) at 110 and R (arginine) at 118 are the amino acid residues derived from the susceptible strain J150. (**B** and (**C**) Western blot of recombinant NSD-1 in Sf9 cells using anti-His antibody (**B**) and anti-NSD-1 antibody (**C**). The membrane fraction was extracted from Sf9 cells and analyzed by western blot. The primary antibodies were anti-His antibody (1:3000) and anti-NSD-1 antibody (1:10,000), respectively. Lane 1, NSD-1-His-Res-nonspl-AcMNPV; 2, NSD-1-His-Sus-nonspl-AcMNPV; 3, Bac1-AcMNPV (negative control); 4, NSD-1-His-Res-spl-AcMNPV; 5, NSD-1-His-Sus-spl-AcMNPV. (**D**) Immunostaining of Sf9 cells infected with recombinant baculoviruses. The Sf9 cells were incubated with anti-NSD-1 antibody (1:100) followed by a secondary antibody labeled with AlexaFluor488 (1:500) (green) and counterstained with DAPI (1:1000) (red). DAPI staining (left); NSD-1 signals (center); merge (right). (**E**) Hypothetical membrane orientation of NSD-1 predicted by SOSUI (http://harrier.nagahama-i-bio.ac.jp/sosui/). The spliced form of NSD-1 lacks the gray region. The red filled circles indicate the positions of amino acids that vary between the resistant (p50T) and susceptible (J150) strains. The green and blue filled circles indicate the putative *N*-linked and *O*-linked glycosylation sites, respectively.

To determine the genomic region responsible for the *nsd-1* phenotype, we performed a genetic linkage analysis using the SNP linkage map^21^ and the *Bombyx* genome database^22^ (Table S1). First, we roughly mapped the *nsd-1-*linked region between two SNP markers, BET089J19 and BET605G10, using 187 BC_1_ individuals (Fig. 1A). Through further mapping, we delimited the locus to an approximately 400-kb-long region on the scaffold Bm_scaf7. KAIKObase search showed that this region contained five candidate genes (Fig. 1B and Table S2 and S3). RT-PCR experiments revealed that three of the five genes, *BGIBMGA001389*, *BGIBMGA001390*, and *BGIBMGA001597*, were expressed in the midgut, where BmDV infects and replicates^2,23^; however, the size of the PCR fragments obtained from each gene was not apparently different between resistant (C124 and p50T) and susceptible strains (J124 and J150) (Fig. 1C). We then determined the full-length cDNA sequences of these three candidate genes from resistant and susceptible strains, and found that *BGIBMGA001597*, but not *BGIBMGA001389* and *BGIBMGA001390*, contained two nucleotide substitutions, both of which resulted in amino acid changes (Figs 2A and S2). Based on these results, we concluded that *BGIBMGA001597* is the best candidate for the *nsd-1* gene (hereafter referred to as *nsd-1*).

### Characterization of the NSD-1 protein

We cloned two variant forms of the *nsd-1* gene using midgut cDNA: a 468-bp nonspliced form that potentially encodes a protein of 155 amino acids and a 393-bp splice variant that potentially encodes a protein of 130 amino acids (Figs 2A and S3). The deduced amino acid sequence from both forms of the *nsd-1* gene did not show high similarity to any known or hypothetical proteins (low alignment score and identity) (Tables S4 and S5), including those of other lepidopteran insects with sequenced genomes^24–28^. The NSD-1 protein was predicted to have a single putative transmembrane domain and a mucin-like domain with a long serine/threonine-enriched stretch (Fig. 2A). Sequence analysis also suggested the presence of *N*- and *O*-linked glycans in this protein (Fig. 2A).

To determine the biochemical properties and topology of NSD-1, we generated several recombinant baculoviruses expressing the nonspliced and spliced forms of NSD-1 with a His-tag. Western blot analyses of proteins from Sf9 cells infected with NSD-1-His-AcMNPV demonstrated that a His-tagged NSD-1 was detected as a 20–40 kDa protein in the membrane fraction of Sf9 cells (Fig. 2B). Also we generated a rabbit polyclonal antibody against bacterially expressed NSD-1, and verified that this anti-NSD-1 antibody can react with NSD-1 (Fig. 2C). According to the deduced amino acid sequence, the masses of the nonspliced and spliced forms are 17 and 14.5 kDa respectively, suggesting that the NSD-1 protein undergoes post-translational modifications.

We next performed immunostaining of Sf9 cells infected with recombinant baculoviruses without permeabilization. As shown in Fig. 2D, we observed that the cell surface was clearly stained with anti-NSD-1 antibody, which is raised against the C-terminal domain of NSD-1. These results indicated that the C-terminus of NSD-1 is located in an extracellular domain, and the two amino acid substitutions found in NSD-1 of susceptible strains occur in the extracellular tail (Fig. 2A,D and E).

### Expression of *nsd-1* and localization of its protein product

**Figure 3:**
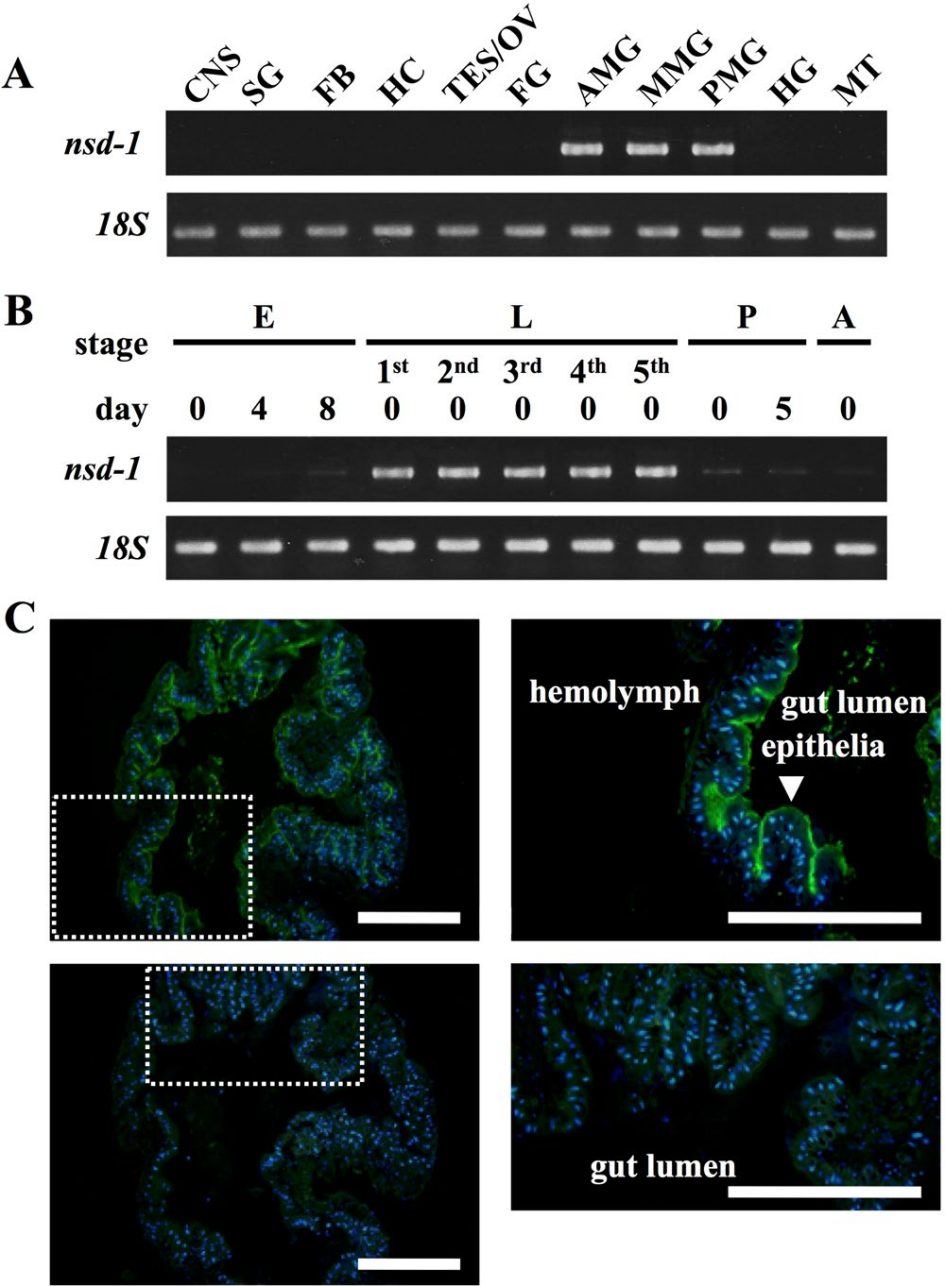
Expression profiles of *nsd-1*. (**A**) Tissue-specific expression of *nsd-1* in a resistant strain, p50T. CNS, central nervous system; SG, silk gland; FB, fat body; HC, hemocyte; TES/OV, testis and ovary; FG, foregut; AMG, anterior midgut; MMG, middle midgut; PMG, posterior midgut; HG, hindgut; MT, Malpighian tubule. Primer sets were designed to amplify *nsd-1* (upper) and ribosomal *18S* (lower) as a positive control. (**B**) Stage-specific expression of *nsd-1* in the resistant strain p50T. E, eggs at on days 0, 4, and 8; L, whole larvae at on day 0 (first to fifth instar); P, whole pupae at on days 0 and 5; A, whole adult at on day 0. (**C**) Immunohistochemical analysis of NSD-1 in the larval anterior midgut. The upper panels show immunofluorescence visualization of NSD-1. The lower panels show control experiments with pre-immune serum. The right panels are zoomed-in images of the dotted boxes in the corresponding left panels. The sections were incubated with anti-NSD-1 antibody (1:200) or pre-immune serum followed by a secondary antibody labeled with AlexaFluor488 (1:200) (green) and counterstained with DAPI (1:1000) (blue). Scale bar: 200 μm.

RT-PCR experiments with total RNA isolated from 11 different tissues showed that *nsd-1* was strongly expressed in all parts of the midgut (Fig. 3A). We also found that *nsd-1* was expressed throughout the larval stage, while the expression level was low in the other stages later than mid-embryogenesis (Figs 3B and S4). Tissue- and stage-specific expression profiles of *nsd-1* were consistent with the unique characteristics of BmDV, which only infects late-embryonic and larval midgut cells^2,29^.

To examine the localization of NSD-1 in the larval midgut, we performed immunohistochemical experiments with the anti-NSD-1 antibody. Immunohistochemical experiments showed that strong NSD-1 signals were detected on the surface of midgut epithelial cells, while no signal was detected in the controls treated with the corresponding pre-immune serum (Fig. 3C). These results indicate that the NSD-1 protein is specifically localized at the surface of midgut epithelial cells.

### Restoration of virus susceptibility by germline transformation of the susceptibility gene + ^*nsd-1*^

To investigate whether the two susceptibility-associated substitutions in NSD-1 are common in other silkworm strains, we determined the *nsd-1* cDNA sequences in 13 resistant and 12 susceptible strains. One of the two residues, arginine 118 (arginine 93 in the spliced form), was conserved in all susceptible strains, while this residue was substituted with other amino acids in the resistant strains (Table S6). This strongly suggests that a single amino acid residue, arginine 118 (93), is crucial for determining viral susceptibility.

**Figure 4:**
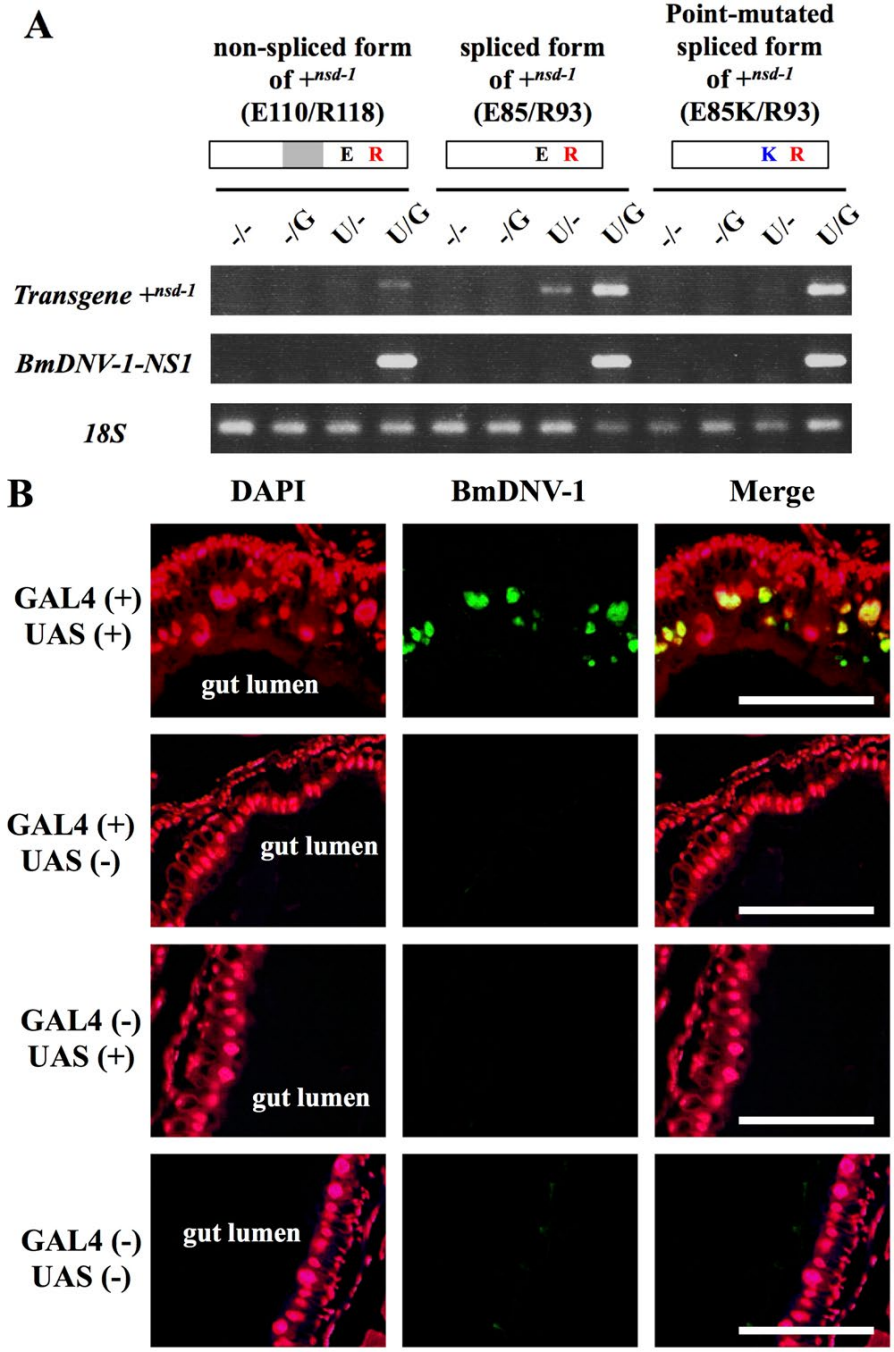
Expression and immunohistochemical analysis in transgenic silkworms. (**A**) RT-PCR of BmDV-derived transcripts and the + ^*nsd-1*^ transgene in the midgut of transgenic silkworms. Transgenic silkworms carrying the nonspliced form of the susceptibility gene + ^*nsd-1*^ [glutamic acid (E) at 110 and arginine (R) at 118], the spliced form of + ^*nsd-1*^ (E at 85 and R at 93), and the point mutated spliced form of + ^*nsd-1*^ [lysine (L) at 85 and R at 93] were generated. K (blue) and R (red) indicate the amino acid residue conserved in resistant and susceptible strains, respectively. −/−, −/G, U/−, and U/G indicate the wild-type, GAL4, UAS, and GAL4/UAS lines, respectively. The midgut was dissected from day 3 fifth instar following exposure to BmDV on day 0 of the fourth instar stage. RT-PCR was performed for 30 cycles with primer sets for + ^*nsd-1*^, the NS1 gene of BmDV^20^, and ribosomal *18S* as a positive control. (**B**) Immunohistochemical analysis of BmDV in the midgut of transgenic silkworms expressing the nonspliced form of the susceptibility gene + ^*nsd-1*^. Sections were incubated with anti-BmDV capsid antibody (1:100) followed by a secondary antibody labeled with AlexaFluor488 (1:200) (green) and counterstained with DAPI (1:1000) (red). DAPI staining (left); BmDV signal (center); merge (right). Scale bar: 200 μm.

To verify that the mutation in *nsd-1* was the sole cause of resistance to BmDV, the nonspliced and spliced forms of the susceptibility gene + ^*nsd-1*^ were introduced into the resistant strain using the GAL4-UAS system^30^. We established transgenic strains expressing + ^*nsd-1*^ under an upstream activating sequence (UAS) together with EGFP as a selectable marker (UAS line). We crossed this UAS line with a previously established GAL4 driver strain carrying DsRed2 (52-2-1 and 193-2) (GAL4 line), and obtained four transgenic progeny lines: GAL4/UAS line (*nsd-1*/*nsd-1*; GAL4/UAS- + ^*nsd-1*^), GAL4 line (*nsd-1*/*nsd-1*; GAL4/−), UAS line (*nsd-1*/ *nsd-1*; −/UAS- + ^*nsd-1*^), and wild-type (*nsd-1*/ *nsd-1*; −/−). We next tested the susceptibility of four transgenic progeny lines after inoculation with BmDV at the first or fourth instar. Although all the lines completed their life cycles without apparent symptoms, we noticed signs of epithelial cell degeneration in the midgut of GAL4/UAS lines at day 3, fifth instar. Therefore, we performed RT-PCR analysis to determine whether the transgenic silkworms were infected with BmDV. BmDV-derived transcript was detected only in the midgut of GAL4/UAS lines that expressed the nonspliced or spliced form of + ^*nsd-1*^ (Fig. 4A). Although the *nsd-1* transcript was faintly detected in the midgut of some of the UAS line, the virus did not replicate (Fig. 4A). The expression level of + ^*nsd-1*^ may have been too low to cause larval death. Also, BmDV transcripts were clearly detected in the midgut cells transformed with nonspliced or spliced *nsd-1* (Fig. 4A). To examine whether a single amino acid residue (arginine 93) could restore viral susceptibility, we next generated transgenic silkworms expressing the point-mutated spliced form of + ^*nsd-1*^. We obtained the same result as that observed in silkworms expressing the nonspliced or spliced form of + ^*nsd-1*^ (Fig. 4A), demonstrating that arginine substitution at a single residue of NSD-1 is sufficient to confer BmDV susceptibility in the resistant strain. Furthermore, immunohistochemical experiments using anti-BmDV capsid antibody confirmed that BmDV propagated in the nuclei of the midgut epithelial cells of the GAL4/UAS line but not in the GAL4 or UAS line (Fig. 4B). These results proved that *nsd-1* controls resistance to the virus, and the mucin-like membrane protein encoded by + ^*nsd-1*^ is required for BmDV infection.

## Discussion

In this study, we molecularly identified the *nsd-1* gene, which potentially encodes a mucin-like single-pass membrane protein. The cDNA sequence of *nsd-1* did not show similarity to any deposited sequences other than cDNA and genome sequences derived from *B. mori* (Tables S4 and S5), suggesting that *nsd-1* is a gene unique to *Bombyx*. In addition, the existence of SNPs in this gene between resistant and susceptible strains and the germline transformation experiments indicated that a single amino acid substitution in the extracellular tail of the NSD-1 protein is sufficient for susceptibility to BmDV (Fig. 4 and Table S6).

There have been many reports on the host factors involved in infection by members of *Parvovirinae*, which belong to the same family as *Densovirinae*. Human parvovirus B19, the best-studied parvovirus, is known to utilize three receptors: erythrocyte P antigen, α5β1 integrin, and Ku80 autoantigen^31–33^. On the other hand, although many histopathological studies on *Densovirinae* infection mechanisms have been conducted using recombinant viruses^34^, only a few host or virus factors required for the infection have been identified thus far^4,34^. In this study, we identified a novel virus resistance gene, *nsd-1*, the protein product of which is a putative cell-surface receptor for BmDV. In addition, we are currently attempting to isolate another BmDV resistance gene, *Nid-1*, by positional cloning. *Nid-1* was shown to be required for a virus infection step that occurs after NSD-1 functions^20^. Based on this and our present results, the product of *Nid-1* may function within the midgut cells. Cloning of *Nid-1* will clarify the intracellular interactions between host and viral gene products during densovirus infection.

Infection mechanisms have been reported for the canine parvovirus (CPV) and the feline panleukopenia virus (FPV). The infection cycle begins with the binding of each virus to the transferrin receptor of the respective host^35^. The relationship between each virus and its host is specific; amino acid residues of the viral capsid protein are recognized by the apical domain of a receptor, which results in a specific binding interaction^35^. In this study, we found that a single amino acid residue of a midgut membrane protein determines the susceptibility phenotype. The positively charged guanidine group of arginine may play an important role in the binding of the BmDV capsid to midgut epithelial cells. Further biochemical studies using recombinant NSD-1 and capsid proteins will contribute to our understanding of the interaction between the receptor and the virus and will allow for the identification of critical residues in the BmDV capsid.

In the present study, the transgenic silkworms did not die after inoculation with BmDV. The immunohistochemical analysis clearly showed that BmDV was propagated in a limited number of midgut cells (Figs 4B and S5). This implies that the low susceptibility of the transgenic silkworms may be because of partial expression of transduced + ^*nsd-1*^ in the midgut cells. The resistance of *B. mori* larvae to BmDV is controlled by two different genes: *nsd-1* and *Nid-1*^15,16^. A study of BmDV infection in silkworms with mosaic midguts showing susceptibility ( + ^*Nid-1*^/ + ^*Nid-1*^) and nonsusceptibility (*Nid-1*/ + ^*Nid-1*^) genotypes demonstrated that columnar cells were degraded by virus infection only in the longitudinal half of the midgut, and surprisingly the larvae did not die^36^. These results indicate that the pathogenicity of BmDV depends on the proportion of infected cells, strongly supporting the low susceptibility phenotype observed in the transgenic silkworms.

Mucins are high-molecular-weight *O*-linked glycoproteins that are either secreted or membrane-bound^37–39^. In general, secreted mucins play roles in hydration, lubrication, and cytoprotection of epithelial cells, whereas membrane-bound mucins function as cell-surface receptors for pathogens and subsequently activate intracellular signaling pathways. Previous reports have shown that T-cell Ig and mucin domain 1 (TIM-1) functions as a receptor for *Zaire ebolavirus*^40^ and hepatitis A virus^41^. Taken together, our data strongly suggest that a newly identified membrane-bound mucin, NSD-1, has a crucial role as a BmDV entry receptor in the silkworm.

In the infectivity of BmBDV and BmDV against *Bombycinae*, *B. mandarina*, which is considered to have a common ancestor to *B. mori*^23^, is susceptible to BmBDV^19^. Native (ancient) strains of *B. mori* also tend to be susceptible; however, these susceptibility strains including *B. mandarina* become resistant to BmDV^42^. This suggests that BmBDV is likely to be the native disease of *Bombyx*, whereas the origin of BmDV is likely to be from other insects. Interestingly, BmBDVs have been isolated in Japan (Yamanashi isolate)^43^, China (Chinese Zhenjiang isolate)^44^, and India (Indian isolate)^45^ so far, whereas BmDV has been found only in Japan^2^. Therefore, the origin of BmDV may be an insect which inhabits in Japan. Cotmore *et al*. reported that all viruses classified into *Iteradensovirus* to date have been isolated from lepidopteran insects^12^, suggesting that a certain lepidopteran insect living in Japan may be the real host of BmDV. Previous studies showed that BmDV was isolated from the mulberry pyralid, *Glyphodes pyloalis*, which is a pest of the Japanese mulberry plantation^46^; however, there is no report about the reproducibility of this result. Therefore, the identification of the real host of BmDV will provide important information on the birth and transmission route of BmDV.

## Methods

### Silkworm strains, virus infection, and cell lines

The silkworm strains resistant to BmDV were B (Hokkaido University), C108T, p50T (The University of Tokyo), C124, No. 104, No. 115, No. 138, No. 141, No. 603, No. 902, and No. 910 (National Institute of Agrobiological Sciences, NIAS); the susceptible strains were J124, J150, No. 101, No. 102, No. 122, No. 123, No. 126, No. 133, No. 142, No. 144, No. 146, and No. 918 (NIAS). *B. mandarina* (Tsukuba native) was also used as resistant species. For linkage analysis, two kinds of single-pair backcrosses (BC_1_) between p50T females and F_1_ males (p50T female × J124 male) and between p50T females and F_1_ males (J150 female × p50T male) were used. All silkworms were reared at 25 °C.

BmDV was prepared as described previously^47^. The newly hatched first instar or the newly ecdysed fourth instar were fed mulberry leaves smeared with a BmDV suspension diluted (at 10^−2^) with the supernatant of a midgut homogenate obtained from BmDV-infected larvae.

The Sf9 cells were cultured at 27 °C in TC-100 (AppliChem) with 10% fetal bovine serum (AppliChem)^48^.

### Positional cloning

For positional cloning, SNP and PCR markers were generated at various positions on linkage group 21, and the markers that were polymorphic between the parents were used for linkage analysis of 1941 BC_1_ larvae selected with virus inoculation from approximately 4000 individuals. The primer sequences used in this study are shown in Table S1. The candidate genes in the region narrowed by linkage analysis were annotated using KAIKObase (http://sgp2010.dna.affrc.go.jp/KAIKObase/).

### Isolation of genomic DNA and total RNA

Genomic DNA was prepared from a small portion of the body (e.g., the caudal end of the abdomen) of the fifth instar using DNAzol (Invitrogen). Total RNA was purified with Trizol (Invitrogen) and subsequently reverse-transcribed with an oligo (dT)^12–18^ primer (GE Healthcare) and Ready-to-Go RT-PCR Beads (GE Healthcare). Full-length cDNAs were cloned using the SMART RACE cDNA Amplification Kit (Clontech).

### Prediction of NSD-1 motifs

NCBI-blastp (http://blast.ncbi.nlm.nih.gov/Blast.cgi), Pfam (http://pfam.sanger.ac.uk/), and SOSUI (http://harrier.nagahama-i-bio.ac.jp/sosui/) were used to search for motifs in the NSD-1 sequence.

### Construction of recombinant baculoviruses

Recombinant Autographa californica multiple nucleopolyhedroviruses (AcMNPVs) were constructed using a Bac-to-Bac system (Invitrogen), as described previously^49^. The coding region of *nsd-1* with a His-tag at the C-terminus was PCR-amplified with a set of gene-specific primers (Table S1) containing restriction enzyme recognition sites. The *nsd-1* fragments were then cloned into a vector, pFastBac1 (Invitrogen), and the bacmids containing the *nsd-1* were isolated. Recombinant AcMNPVs, NSD-1-His-tag-resistance-type-nonspliced form-AcMNPV (NSD-1-His-Res-nonspl-AcMNPV), NSD-1-His-tag-susceptible-type-nonspliced form-AcMNPV (NSD-1-His-Sus-nonspl-AcMNPV), NSD-1-His-tag-resistance-type-spliced form-AcMNPV (NSD-1-His-Res-spl-AcMNPV), and NSD-1-His-tag-susceptible-type-spliced form-AcMNPV (NSD-1-His-Sus-spl-AcMNPV), were generated by bacmid transfection into Sf9 cells, and used in western blotting and immunostaining analysis. The parental virus, Bac1-AcMNPV^49^, was used as the control.

### Preparation of recombinant NSD-1 in insect cells

Sf9 cells (4 × 10^6^ cells seeded in a 60 mm cell culture dish) were infected with 150 μl of P2 stock virus solution of recombinant AcMNPVs, NSD-1-His-Res-nonspl-AcMNPV, NSD-1-His-Sus-nonspl-AcMNPV, NSD-1-His-Res-spl-AcMNPV, NSD-1-His-Sus-spl-AcMNPV, and Bac1-AcMNPV (negative control). At 72 hours post infection (hpi), infected cells were collected. The cells were rinsed with PBS, and homogenized twenty times in 400 μL of PBS in the presence of a cocktail of proteinase inhibitors (Roche) using the tight-fitting pestle. The homogenized cells were centrifuged at 800 ×*g* for 10 min. The resulting microsomal supernatant was spun at 20,500 × *g* for 30 min at 4 °C, and the resulting pellet was collected as the membrane protein fraction.

### Western blot analysis

Western blot analysis was performed as described previously^50^. After SDS-PAGE, the proteins were blotted onto a polyvinylidene fluoride (PVDF) membrane (Immobilon-P, Millipore) using a blotting apparatus (ATTO) in a transfer buffer (25 mM Tris, 192 mM glycine, 20% methanol). The membrane was incubated in TBS-T buffer (20 mM Tris-HCl pH 8.0, 150 mM NaCl, and 0.05% Tween 20) containing 4% *Blockace* (*Dainihon* Pharmaceutics) followed by 1 h of incubation at room temperature with anti-His (Qiagen, 1:3000), anti-NSD-1 (1:10,000) dilution in TBS-T containing 1% *Blockace*. The blot was washed three times with TBS-T buffer for 5 min and subsequently incubated with goat anti-mouse or rabbit horseradish peroxidase-conjugated secondary antibody (Zymed laboratory, 1:3000) dilution in TBS-T containing 1% *Blockace* for 1 h at room temperature. The signals were detected with an Immobilon Western Chemiluminescent HRP Substrate (Millipore) and visualized using the LAS1000 Plus Imaging System (Fuji Film). To detect the NSD-1 protein, we generated rabbit polyclonal antibodies against a recombinant protein corresponding to the extracellular domain of NSD-1. In brief, PCR-amplification products (ca. 0.3 kbp) corresponding to amino acids 42–155 of NSD-1 (GenBank accession No. AB546955) were digested with the restriction enzymes (Table S1), inserted into the pET24b vector (Novagen), and introduced into *Escherichia coli* BL21 (DE3) competent cell (BioDynamics Laboratory Inc). NSD-1 expression was induced at 37 °C in the presence of 1 mM isopropyl-1-thio-β-D-galactopyranoside induction. The soluble fraction including the recombinant protein was purified using a HisGraviTrap column (GE Health-care Bioscience) according to the manufacturer’s protocol. The purified recombinant proteins (ca. 15 kDa) were used to generate anti-NSD-1 rabbit polyclonal antibody (Operon, Tokyo, Japan).

### Immunohistochemistry

Sf9 cells (5 × 10^5^ cells seeded in a 35 mm cell culture dish) were infected with 100 μl of P2 stock virus solution of recombinant baculoviruses. At 72 hpi, infected cells were washed three times with PBS, and fixed in 1% glutaraldehyde/PBS (Nacalai tesque) for 10 min. The cells were washed three times with TBS-T, and blocked overnight at 4 °C using TBS-T containing 4% *Blockace*. Next the cells were incubated with the anti-NSD-1 antibody (1:100) dilution in TBS-T containing 1% *Blockace* for 1 h, and subsequently washed three times with TBS-T for 5 min. The cells were incubated with AlexaFluor488-labeled goat anti-rabbit IgG F(ab)_2_ fragment (Invitrogen, 1:500) dilution in TBS-T containing 1% *Blockace* for 1 h, and subsequently washed three times with TBS-T for 5 min. The cells were subsequently stained with 4′,6-diamidino-2-phenylindole dihydrochloride (DAPI) (DOJIN, 1:1000), and examined by FLoid™ Cell Imaging Station (Life technologies) as described previously^51^.

Immunohistochemical studies using midgut sections were conducted as described previously^50^. Sections of the anterior part of the midgut were obtained from day 3 fifth instar p50T (resistant strain) and from fourth instar transgenic silkworm, which were inoculated with BmDV one week prior to this experiment. The primary antibodies used were anti-NSD-1 (1:200) or anti-BmDV capsid (1:100), and the secondary antibody was an AlexaFluor488-labeled goat anti-rabbit IgG F(ab)_2_ fragment (1:200). The slides were counterstained with DAPI (1:1000). Pre-immune serum was also used as a control. To detect BmDV, we generated rabbit polyclonal antibodies against recombinant protein corresponding to the BmDV capsid protein (GenBank accession No. AAK55490, the coding region corresponding to amino acids 365–673). Anti-BmDV rabbit polyclonal antibody was produced by the same method as that of anti-NSD-1 rabbit polyclonal antibody. The primer sequences used in this study are shown in Table S1.

### Generation of transgenic silkworms

The stable transgenic strains using the binary GAL4/UAS system were generated as described previously^19^. For the activator strain, two GAL4 lines that have been shown to direct transgene transcription in the midgut were selected: 52-2-1 and 193-2 (genotype, *nsd-1*/*nsd-1*; line, GAL4/−). To prepare the effector plasmid (for UAS line), the nonspliced and spliced forms of the susceptibility gene ( + ^*nsd-1*^) were amplified by PCR from the cDNA clone, and the PCR products were cloned into pBacMCS-UAS.SV40-3xP3EGFP. The effector construct containing the UAS- + ^*nsd-1*^ sequence and the transposase-carrying helper plasmid, pHA3PIG^30^, were injected into preblastoderm *w1-pnd* ( + ^*nsd-1*^/ + ^*nsd-1*^; −/−) embryos at a concentration of 0.2 mg/ml.

After sibling selection with the marker, EGFP, G_1_ moths of the UAS line ( + ^*nsd-1*^/ + ^*nsd-1*^; −/UAS- + ^*nsd-1*^) were crossed with moths (*nsd-1*/*nsd-1*; −/−). Subsequently, the G_2_ moths (*nsd-1*/ + ^*nsd-1*^; −/UAS- + ^*nsd-1*^) were crossed with moths of the GAL4 line (*nsd-1*/*nsd-1*; GAL4/−) to generate + ^*nsd-1*^-overexpressing GAL4/UAS lines (*nsd-1*/*nsd-1*; GAL4/UAS- + ^*nsd-1*^).

To construct plasmids containing point mutations, two-step PCR-based mutagenesis was performed using the four cDNA constructs described above as templates^50^. The primers used in the mutagenesis experiments are shown in Table S1.

### Data availability

The datasets generated from this study supporting our finding are available from the corresponding author on reasonable request.

### Accession codes

The nucleotide sequences reported in this paper have been deposited in the DNA database of GenBank (accession Nos. AB546955, AB546956, AB546957, and AB546958).

## Electronic supplementary material

supplementary information
