# Supplementary material for: A single amino acid substitution in the Bombyx-specific mucin-like membrane protein causes resistance to Bombyx mori densovirus
Source: Sci Rep. 2018 May 9;8:7430. doi: 10.1038/s41598-018-25388-7 (PMC5943349; doi:10.1038/s41598-018-25388-7)
Supplement: Supplementary file 1 — supplementary information [file 41598_2018_25388_MOESM1_ESM.pdf]

A single amino acid substitution in the *Bombyx*-specific mucin-like membrane protein causes resistance to *Bombyx mori* densovirus

Katsuhiko Ito[1, 2, 3*], Kurako Kidokoro[1], Susumu Katsuma[2], Hideki Sezutsu[4, #a], Keiro Uchino[4, #a], Isao Kobayashi[4, #a], Toshiki Tamura[4, #a], Kimiko Yamamoto[1, #b], Kazuei Mita[1, #c], Toru Shimada[2], and Keiko Kadono-Okuda[1*, #d]

[1]Insect Genome Research Unit, National Institute of Agrobiological Sciences, Tsukuba, Ibaraki 305-8634, Japan, [2]Department of Agricultural and Environmental Biology, Graduate School of Agricultural and Life Sciences, The University of Tokyo, Bunkyo-ku, Tokyo 113-8657, Japan, [3]Department of Science of Biological Production, Graduate School of Agriculture, Tokyo University of Agriculture and Technology, Fuchu, Tokyo 183-8509, Japan, [4]Transgenic Silkworm Research Unit, National Institute of Agrobiological Sciences, Tsukuba, Ibaraki 305-8634, Japan.

[#a]Current Address: Transgenic Silkworm Research Unit, Institute of Agrobiological Sciences, National Agriculture and Food Research Organization, Tsukuba, Ibaraki 305-8634, Japan.

[#b]Current Address: Division of Applied Genetics, Institute of Agrobiological Science, National Agriculture and Food Research Organization, Tsukuba, Ibaraki 305-8634, Japan.

[#c]Current Address: State Key Laboratory of Silkworm Genome Biology, Southwest University, Chongqing, 400715, China.

[#d]Current Address: Division of Biotechnology, Institute of Agrobiological Sciences, National Agriculture and Food Research Organization, Tsukuba, Ibaraki 305-8634,

Japan.

*Corresponding authors:

Katsuhiko Ito, Department of Science of Biological Production, Graduate School of Agriculture, Tokyo University of Agriculture and Technology, 3-5-8 Saiwai-cho, Fuchu, Tokyo 183-8509, Japan. Tel&Fax: +81-42-367-5786. E-mail: katsuito@cc.tuat.ac.jp

Keiko Kadono-Okuda, Division of Biotechnology, National Agriculture and Food Research Organization, 1-2 Owashi, Tsukuba, Ibaraki 305-8634, Japan. Tel&Fax: +81-29-838-6102. E-mail: kadono@affrc.go.jp

Supplementary information, Figure S1

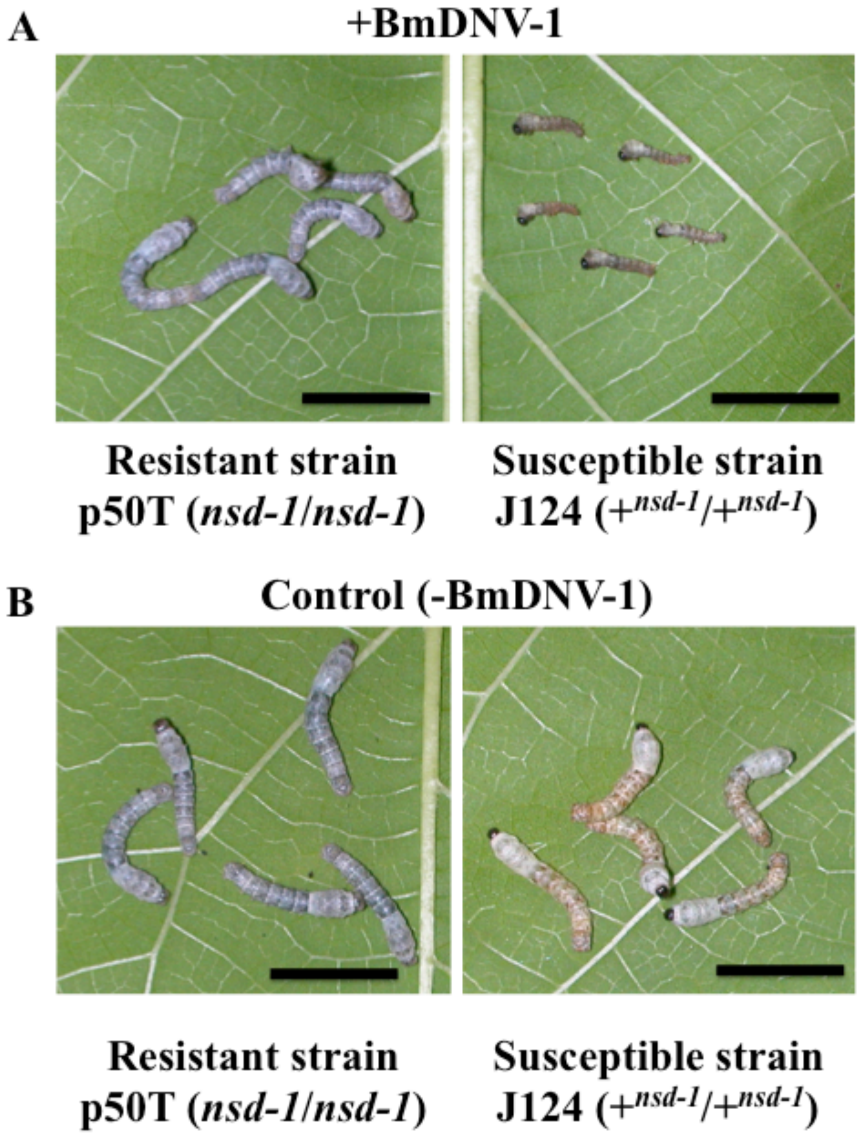

Supplementary Figure 1. Comparison of resistant and susceptible strains after BmDNV-1 inoculation and non-inoculated control. (A) Resistant p50T (*nsd-1*/*nsd-1*) (left) and susceptible J124 ($+^{nsd\text{-}1}/+^{nsd\text{-}1}$) (right) after BmDNV-1 inoculation. Larvae were inoculated with BmDNV-1 at day 0 of first instar. (B) Resistant p50T (*nsd-1*/*nsd-1*) (left) and susceptible J124 ($+^{nsd\text{-}1}/+^{nsd\text{-}1}$) (right) without inoculation. Scale bar: 10 mm.

Supplementary information, Figure S2

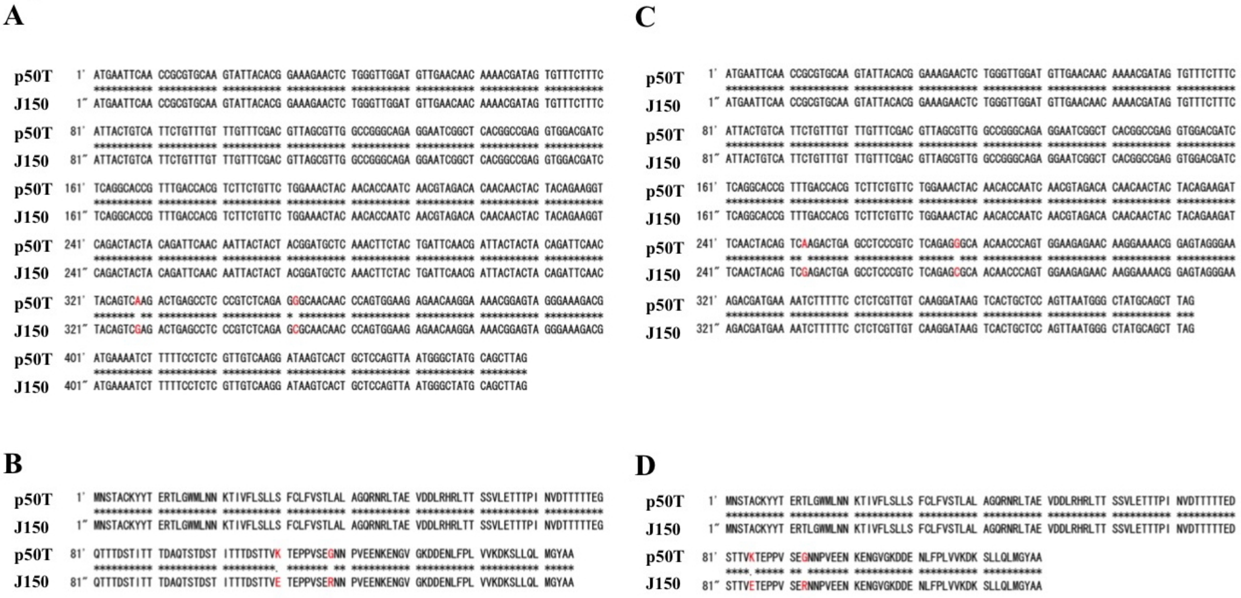

Supplementary Figure 2. Sequence alignments of *nsd-1*. (A, B) Alignment of nucleotide (A) and deduced amino acid (B) sequences of the non-spliced form of the *nsd-1* gene from resistant (p50T) and susceptible (J150) strains. (C, D) Alignment of nucleotide (C) and deduced amino acid (D) sequences of the spliced form of the *nsd-1* gene from p50T and J150. The red characters indicate nucleotide and amino acid substitutions between p50T and J150.

Supplementary information, Figure S3

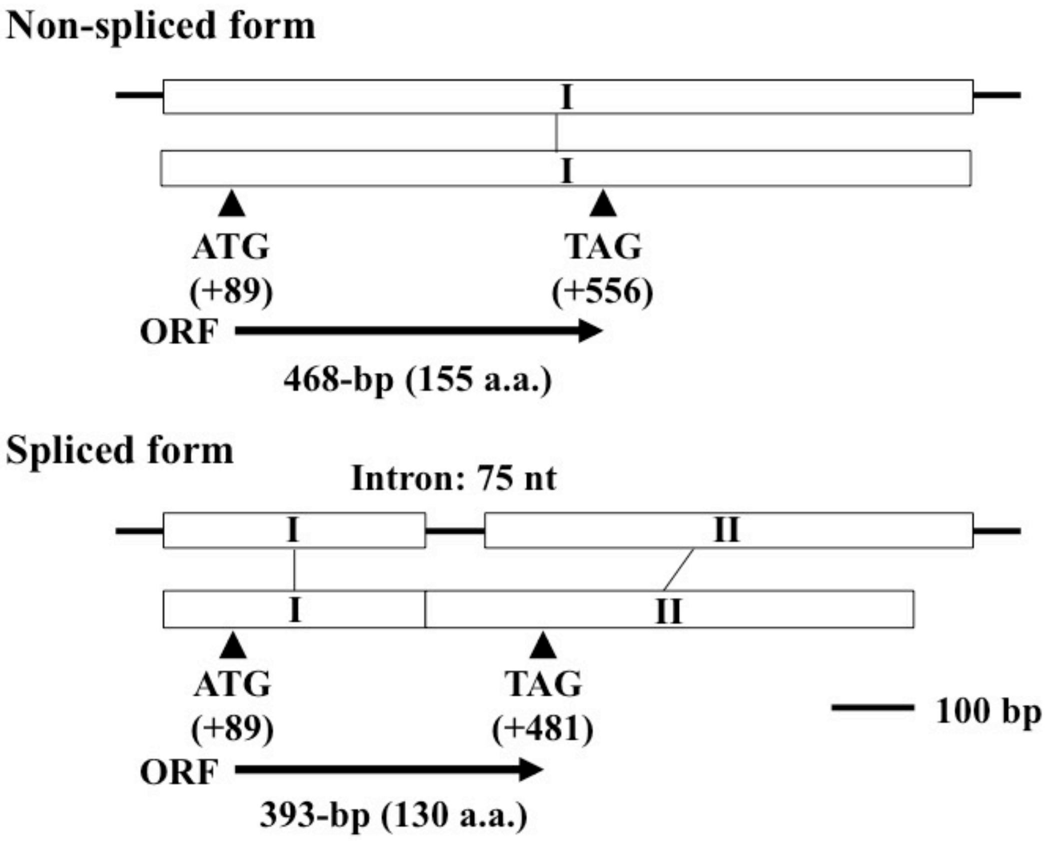

Supplementary Figure 3. Schematic structure of genomic and cDNA of *nsd-1* in the resistant strain p50T. The upper and lower panels show non-spliced and spliced forms of *nsd-1*, respectively. The arrowheads show the positions of start and stop codons. The arrows indicate the coding regions.

Supplementary information, Figure S4

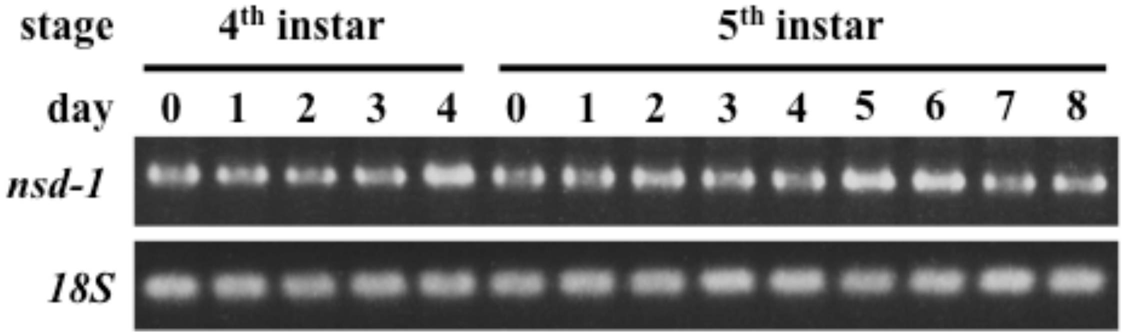

Supplementary Figure 4. Stage-specific expression of *nsd-1*. RT-PCR analysis of the *nsd-1* gene in the resistant strain p50T. From the left to the right; midgut of fourth (days 0–4), and fifth (days 0–8) instar silkworms.

Supplementary information, Figure S5

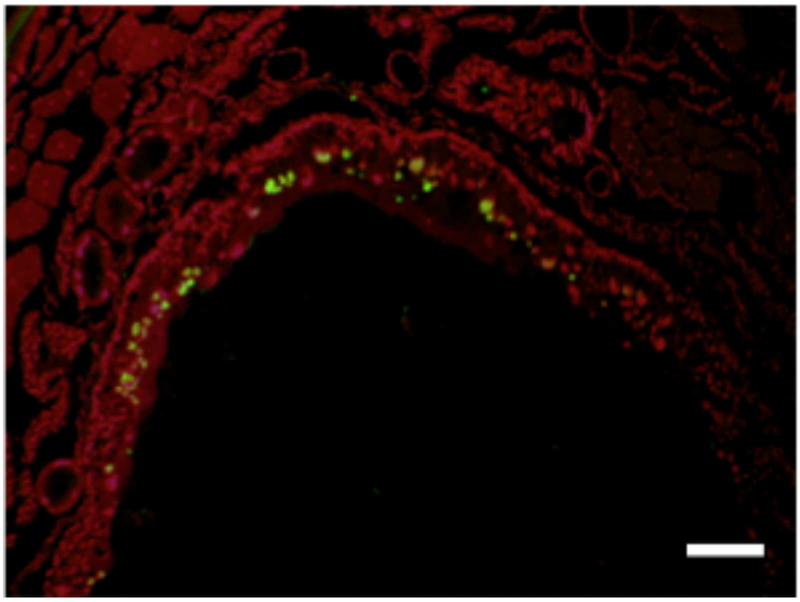

Supplementary Figure 5. Localization of BmDNV-1 in the midgut cells of transgenic silkworms. Immunohistochemical analysis of BmDNV-1 in the midgut of transgenic silkworms expressing the non-spliced form of the susceptibility gene $+^{nsd\text{-}1}$ was performed. The sections were incubated with anti-BmDNV-1 capsid antibody (1:100), followed by a secondary antibody labeled with AlexaFluor488 (1:200) (green), and counterstained with DAPI (red). Scale bar: 200 μm.

Supplementary information, Table S1 The primers used in this research

object	name	sequence (5' - 3' end)	Restriction enzyme
Linkage analysis in SI Table 2	T059O10-F	GATAGTGGTGCGCGTGGT	
	T059O10-R	GACGACGAGCCGTGGTAG	
	T024K22-F	GCAAAAGCCATTATTCTATTTTCA	
	T024K22-R	TGAACAAAAGTAATTTAATAATAGCCC	
	T018B21-F	GTCTACGGAAGCGTCAAAGC	
	T018B21-R	GCGAAGCACGAATCTTCTTT	
	Sc14272-89J19T-F	TTTATTTCAGCGGATTCGGTATTTG	
	Sc14272-89J19T-R	TTTAATTGGATCCACGGTATTGGAA	
	Sc3970-52D14T-F	GACACTAATGGGTTCGTTCATGTCC	
	Sc3970-52D14T-R	TTTTGCACCTTTCTTTTGTTTGTTGA	
	Sc8241-62B09T-CF	TAAGAACATTAAGAAAGCCTTAATGC	
	Sc8241-62B09T-CR	GCCCTTTATTTATGTTTAGGTTACC	
	T062B09-F	TTCGCTTATGTGTCCGTGAA	
	T062B09-R	GGACCACGCCTATCACACC	
	Sc6678-09B04T-F	GGGTGAGGTCCGTTTAAATCAAATACT	
	Sc6678-09B04T-R	CCTGCCCATTACTTTTGAGACTTCA	
	BGIBMGA001597-F	TGCAAGTATTACACGGAAAGAACTC	
	BGIBMGA001597-R	AAGTGACCAATCGTGTAGTATTACG	
	Sc10109-48J16S-F	GACACTGGATGTTAAGGGATCACCA	
	Sc10109-48J16S-R	CTCGCTATCTAGGTGCACGAACAAT	
	T605G10-F	TGTTTTGGACATATCCTGCG	
	T605G10-R	CATCCGTGAAAACCACTGTAGA	
	T611D06-F	TTTGTTTTGGACGCGAGATT	
	T611D06-R	TTCGTTCAGGGAATTGATGA	
RT-PCR for *nsd-1* candidate	BGIBMGA001388-F	TCTTCGACAATGAAACTGCAAATCA	
	BGIBMGA001388-R	TTGTTTACGCGTTCGCTCTTCATAC	
	BGIBMGA001389-F	TGATGAAGTTGCAAATCGTTGTGTT	
	BGIBMGA001389-R	TTCATGCGGAGATTGACGAAGTTAT	
	BGIBMGA001390-F	CAGTAGAACCTTCGTTTGACCATCG	
	BGIBMGA001390-R	GGACAGTGATGTAGCCATGAAACCT	
	BGIBMGA001596-F	ATTACTCATGGAGGCGCTAACCAAT	
	BGIBMGA001596-R	GTCACTTCCAGAGATGAACGAGGTC	
	BGIBMGA001597-F	TGCAAGTATTACACGGAAAGAACTC	
	BGIBMGA001597-R	AAGTGACCAATCGTGTAGTATTACG	
	18S-sense	TTGACGGAAGGGCACCACCAG	
	18S-antisense	GCACCACCACCCACGGAATCG	
Plasmid construct for recombinat baculoviruses	NSD-1-pFast-F	**GGATCC**ATGAATTCAACCGCGTGCAAGTATTAC	*Bam*HI
	NSD-1-pFas-Chis-R	**TCTAGA**TTA<u>ATGATGATGATGATGATG</u>AGCTGCATAGCCCATTAACTGGAG	*Xba*I
RT-PCR for *nsd-1* in Figure 3	*nsd-1*-F	TTGGGACGTGCTGGAATATTTATTAGAA	
	nsd-1-R	GATATTGCTTGGTTGTGGCTCGATACT	
Plasmid construct for bacterial expression system	pET-mucin-F	**GGATCC**GGGGCAGAGGAATCGGCTCACG	*Bam*HI
	pET-mucin-R	**CTCGAG**AGCTGCATAGCCCATTAACTGGAG	*Xho*I
	pET-BmDV-capsid-F	CC**GGATCC**GGCTCTTGGAACTGAATGGAAAAATGC	*Bam*HI
	pET-BmDV-capsid-R	CC**CTCGAG**TTTATTTAGTTTACTAGATCTAGTTAC	*Xho*I
Plasmid construct for $+^{nsd\text{-}1}$ transgene	mucin-TG-F	**TCTAGA**ATGAATTCAACCGCGTGCAAGTATTAC	*Xba*I
	mucin-TG-R	**TCTAGA**CTAAGCTGCATAGCCCATTAACTGGAG	*Xba*I
	mucin-TG-mutation-1F	CGTCTCAGAGgGCAACAACCCAG	
	mucin-TG-mutation-1R	CTGGGTTGTTGCcCTCTGAGACG	
Check of the transcripts in the transgenic silkworm in Figure 4	pBacMCS-UAS-2F	GCAACTACTGAAATCTGCCAAG	
	mucin-cds-1R	GCATAGCCCATTAACTGGAGCAG	
	BmDNV-1-NS1-F	CGAGCCGAAATTGGTACATT	
	BmDNV-1-NS1-R	CCTGATCGTTGCTCTCTTCC	

Bold and underlined letters indicate restriction enzyme sites and the His-tag, respectively.

Supplementary information, Table S2 Linkage analysis of BC1 segregants

primer sets	method	p50T	J124 or J150	F_1	BC_1*	
T059O10-F/R	sequence	A	B	A/B	A/B	A
T024K22-F/R	sequence	A	B	A/B	A/B	A
T018B21-F/R	sequence	A	B	A/B	A/B	A
Sc14272-89J19T-F/R	sequence	A	B	A/B	A/B	A
Sc3970-52D14T-F/R	sequence	A	B	A/B	A/B	A
**Sc8241-62B09T-CF/CR**	sequence	A	B	A/B	A/B	A
T062B09-F/R	sequence	A	B	A/B	A	A
Sc6678-09B04T-F/R	PCR	A	B	A/B	A	A
BGIBMGA001597-F/R	sequence	A	B	A/B	A	A
**Sc10109-48J16S-F/R**	PCR	A	B	A/B	A	A/B
T605G10-F/R	sequence	A	B	A/B	A	A/B
T611D06-F/R	sequence	A	B	A/B	A	A/B

'A' indicates p50T homozygous, 'B' indicates J124 or J150 homozygous and 'A/B' indicates heterozygous genotype.

Heterozygous genotypes are shaded.

The bold letters indicate primer sets located most closely to *nsd-1* linked region.

* In linkage analysis, about 2000 BC_1 individuals with the *nsd-1* phenotype were used.

Supplementary information, Table S3 The list of five candidate genes of *nsd-1*

Gene name	chromosome	scaffold	scaffold start position	scaffold end position	Description
BGIBMGA001388	chr21	Bm_scaf7	7563282	7565058	undefined
BGIBMGA001389	chr21	Bm_scaf7	7541390	7543171	undefined
BGIBMGA001390	chr21	Bm_scaf7	7503784	7514319	GO:0005622 intracellular,IPR001357|BRCT
BGIBMGA001596	chr21	Bm_scaf7	7531946	7536363	IPR003736|Phenylacetic acid degradation-related protein, IPR006683|Thioesterase superfamily
BGIBMGA001597	chr21	Bm_scaf7	7739821	7740288	undefined

Supplementary information, Table S4 NCBI blast-p search with the amino acid sequence deduced from nonspliced form of *nsd-1*

Description	Max score	Query cover	E value	Identity	Accession
uncharacterized protein LOC101747082 [Bombyx mori]	254	100%	5.00E-85	84%	XP_004929728.1
Uncharacterized protein OBRU01_07177 [Operophtera brumata]	99	98%	1.00E-23	42%	KOB75644.1
hypothetical protein B5V51_970 [Heliothis virescens]	98.6	33%	5.00E-23	87%	PCG79382.1
PREDICTED: uncharacterized protein LOC106129607 [Amyelois transitella]	90.9	37%	7.00E-20	74%	XP_013183662.1
PREDICTED: ELMO domain-containing protein C-like [Papilio machaon]	70.1	72%	2.00E-12	52%	XP_014357456.1
PREDICTED: uncharacterized protein LOC106104931 [Papilio polytes]	67	96%	2.00E-11	46%	XP_013140584.1
hypothetical protein RR46_06246 [Papilio xuthus]	66.2	32%	7.00E-11	78%	KPJ03088.1
aspartate and glycine-rich protein-like isoform X8 [Pieris rapae]	60.1	34%	2.00E-08	70%	XP_022129265.1
circumsporozoite protein-like isoform X7 [Pieris rapae]	60.1	34%	2.00E-08	70%	XP_022129264.1
circumsporozoite protein-like isoform X5 [Pieris rapae]	60.1	34%	2.00E-08	70%	XP_022129262.1
circumsporozoite protein-like isoform X4 [Pieris rapae]	60.1	34%	2.00E-08	70%	XP_022129261.1
circumsporozoite protein-like isoform X2 [Pieris rapae]	60.1	34%	2.00E-08	70%	XP_022129259.1
circumsporozoite protein-like isoform X1 [Pieris rapae]	60.1	34%	2.00E-08	70%	XP_022129258.1
uncharacterized protein LOC112049504 [Bicyclus anynana]	57.4	35%	2.00E-07	60%	XP_023943173.1

Supplementary information, Table S5 NCBI blast-p search with the amino acid sequence deduced from spliced form of *nsd-1*

Description	Max score	Query cover	E value	Identity	Accession
uncharacterized protein LOC101747082 [Bombyx mori]	265	100%	1.00E-89	100%	XP_004929728.1
Uncharacterized protein OBRU01_07177 [Operophtera brumata]	95.9	97%	8.00E-23	43%	KOB75644.1
hypothetical protein B5V51_970 [Heliothis virescens]	96.7	40%	1.00E-22	87%	PCG79382.1
PREDICTED: uncharacterized protein LOC106129607 [Amyelois transitella]	89	44%	1.00E-19	74%	XP_013183662.1
hypothetical protein RR46_06246 [Papilio xuthus]	71.6	96%	2.00E-13	46%	KPJ03088.1
PREDICTED: uncharacterized protein LOC106104931 [Papilio polytes]	70.1	96%	9.00E-13	46%	XP_013140584.1
PREDICTED: ELMO domain-containing protein C-like [Papilio machaon]	66.2	56%	3.00E-11	65%	XP_014357456.1
aspartate and glycine-rich protein-like isoform X8 [Pieris rapae]	60.1	41%	1.00E-08	70%	XP_022129265.1
circumsporozoite protein-like isoform X7 [Pieris rapae]	60.1	41%	1.00E-08	70%	XP_022129264.1
circumsporozoite protein-like isoform X5 [Pieris rapae]	60.1	41%	1.00E-08	70%	XP_022129262.1
circumsporozoite protein-like isoform X4 [Pieris rapae]	60.1	41%	1.00E-08	70%	XP_022129261.1
circumsporozoite protein-like isoform X2 [Pieris rapae]	60.1	41%	1.00E-08	70%	XP_022129259.1
circumsporozoite protein-like isoform X1 [Pieris rapae]	60.1	41%	1.00E-08	70%	XP_022129258.1
uncharacterized protein LOC112049504 [Bicyclus anynana]	58.9	81%	3.00E-08	41%	XP_023943173.1
PREDICTED: glutamyl aminopeptidase-like [Papilio machaon]	43.9	36%	0.028	40%	XP_014362466.1
PREDICTED: glutamyl aminopeptidase-like isoform X2 [Papilio xuthus]	42.7	32%	0.073	45%	XP_013180265.1
PREDICTED: glutamyl aminopeptidase-like isoform X1 [Papilio xuthus]	42.7	32%	0.073	45%	XP_013180257.1
PREDICTED: glutamyl aminopeptidase-like isoform X1 [Papilio polytes]	42	25%	0.14	52%	XP_013142751.1
hypothetical protein KGM_213708 [Danaus plexippus plexippus]	40.8	34%	0.27	37%	OWR42837.1
heterogeneous nuclear ribonucleo HRP1 protein [Rutstroemia sp. NJR-2017a BVV2]	38.9	56%	1.5	31%	PQE05771.1

Supplementary information, Table S6 Comparison of amino acid between resistant and susceptible strains

strain	position	
	110 (85) a.a*	118 (93) a.a*
Resistant strains		
B	K	G
p50T	K	G
t32	K	G or S
C108T	K	G
C124	K	G
No. 104	K	P
No. 115	K	P
No. 138	K	G
No. 141	K	P
No. 603	K	P
No. 604	K	P or H
No. 902	K	P
No. 910	K	P
*B. mandarina***	K	P
Susceptible strains		
J124	E	**R**
J150	E	**R**
No. 101	E	**R**
No. 102	E	**R**
No. 122	K	**R**
No. 123	K	**R**
No. 126	E	**R**
No. 133	E	**R**
No. 142	K	**R**
No. 144	E	**R**
No. 146	E	**R**
No. 918	K	**R**

*The number indicates the position of amino acid substitution of non-spliced (spliced) form.
E, G, H, K, P, R, and S indicate glutamic acid, glycin, histidine, lysine, proline, arginine, and serine, respectively.
R at 118 (or 93) was conserved in all of the susceptible strains.
***B. mandarina* was collected at Tsukuba (Ibaraki Prefecture).
